# Research on Cracking Mechanism of Early-Age Restrained Concrete under High-Temperature and Low-Humidity Environment

**DOI:** 10.3390/ma14154084

**Published:** 2021-07-22

**Authors:** Min Yuan, Sheng Qiang, Yingli Xu, Yu Li, Wenqiang Xu

**Affiliations:** 1 College of Water Conservancy and Hydropower Engineering, Hohai University, Nanjing 210098, China; miny2018@163.com (M.Y.); yli713@hhu.edu.cn (Y.L.); xwqiang@hhu.edu.cn (W.X.); 2 Department of Water Conservancy Engineering, Zhejiang Tongji Vocational College of Science and Technology, Hangzhou 311231, China; xuyingli@zjtongji.edu.cn

**Keywords:** high-temperature and low-humidity, early-age, restrained, cubic curve model, macrocracks, microstructure

## Abstract

How to prevent the cracking of tunnel lining concrete under a high-temperature and low-humidity environment has gradually become a challenge faced by the engineering community. Actually, the concrete structure will be restrained, which easily leads to cracking. Aiming at this problem, a self-restraint device of concrete specimens was designed in this paper, which aims to more realistically simulate the restrained state of concrete structures during construction. SEM, EDS and XRD detection methods were used to study the macroscopic and microscopic properties of an early-age restrained concrete specimen under a high-temperature and low-humidity environment, and the results were compared with those of a non-restrained concrete specimen. The results show that the change in the internal relative humidity of the concrete was an extremely slow process, and the response rate of the internal humidity of the concrete was much slower than that of the temperature. A cubic curve model was used to fit the measured concrete damage degree with the loading age, and the fitting effect was good. Under the environment of high temperature and low humidity, the loading age from the 0.6th day to the 1st day was the period of a relatively large fluctuation in the concrete temperature and humidity, and the restraint would aggravate the damage of the concrete. The damage degree increased with the increase in the loading age, the microcracks gradually increased and, finally, macrocracks were formed. The restraint effect was to intensify the formation of microcracks, affect the hydration of the cement at the micro level and, finally, increase the risk of concrete cracking perpendicular to the restrained direction at the macro level. The research results may provide guidance for research on the cracking mechanism of tunnel lining concrete constructed under a high-temperature and low-humidity environment.

## 1. Introduction

A large number of engineering practices have proved that 80% of concrete structural cracks are mainly caused by the non-load stress generated by the concrete material itself or under the action of environmental temperature and humidity changes [1]. There will be no stress related to temperature deformation during cooling if the concrete structure can move freely. In the early age of a concrete structure, due to the self-drying effect of cement-based materials [2,3,4] or external drying [5,6], the humidity of the concrete decreases, which leads to drying shrinkage deformation of different parts of the structure. The drying shrinkage of the surface of the structure is restrained by the internal concrete, which will produce considerable tensile stress and easily cause surface cracks [7,8]. If the surface cracks are superimposed on the temperature stress or other harmful factors, they will develop deep cracks, or penetrating cracks [9,10] will occur, which seriously endanger the safety of the structure. Taking the Sichuan–Tibet Railway in the construction of Western China as an example, there are many high-temperature tunnels along the line, and the ground temperature is 28.7 to 89.0 °C. High temperature and low humidity [11,12] are the main forms of tunnel high ground temperature. The rapid heating and dehydration of the concrete after pouring under the action of a high ground temperature will lead to the deterioration of the microstructure of cement-based materials [13,14]. Combined with the combined effect of physical and chemical shrinkage, the performance of concrete will be greatly affected. How to prevent cracks in tunnel lining concrete constructed in a high-temperature and low-humidity environment [15] has gradually become a problem faced by the engineering community, which has attracted close attention.

On the other hand, as a multiphase composite material, concrete will produce initial cracks before external load, namely, initial damage [16], which mainly includes interfacial cracks caused by uneven settlement between the coarse aggregate and mortar [17], and mortar cracks caused by uneven shrinkage of the mortar itself [18]. According to the microscopic mechanism, the microcracks in concrete begin to increase at the beginning of tensile damage. With the increase in the damage degree, the microcracks gradually increase. The performance of concrete materials depends largely on the internal microcracks. Under the action of an external load, the microcracks in concrete will expand and converge, finally forming macrocracks. Macrocracks will cause strength, stiffness and other performance degradations, and even material damage [19,20], namely, material damage. The macroscopic cracks will cause new damage and then accumulate to cause the cracks to expand. This continuous expansion will cause great harm to the stability of the structure.

In fact, the concrete structure will be restrained, either restrained by the external rock base [21], restrained by the deformation difference caused by the temperature gradient inside [22] or restrained by the drying shrinkage of the structural surface by the internal concrete [23]. Restraints will also increase the damage of the concrete structure to a certain extent, and microcracks will further increase, thereby increasing the risk of cracking in the concrete structure. At present, the widely used restrained experimental methods are the temperature stress machine method [24,25], the ring restrained method [26,27], the elliptical ring restrained method [28,29], the axial restrained method [30,31], the plate restrained method [32], etc. However, the above methods have at least one shortcoming, such as a high equipment cost, difficult production or poor operability in a high-temperature environment [33].

Based on the characteristics of the low thermal expansion coefficient and high strength of Invar alloy [34], a concrete specimen restraint device was designed in this paper. The device is simple in structure, low in cost and strong in operability, aiming to more realistically simulate the restrained state of concrete structures during construction. SEM, EDS and XRD were used to study the macroscopic and microscopic properties of the early-age restrained concrete specimen under a high-temperature and low-humidity environment, and the results were compared with those of a non-restrained concrete specimen. The research results may provide guidance for the study of the cracking mechanism of tunnel lining concrete under a high-temperature and low-humidity environment.

## 2. Experiment Program

### 2.1. Materials and Mix Proportion

P·O 42.5 common Portland cement was used, which conformed to the Chinese standard GB175-2007. Natural fine aggregate was used. The fineness modulus of sand was 2.65. The coarse aggregate was granite gravel. The continuous gradation was 5 to 25 mm. Polycarboxylate superplasticizer was used, and the water was ordinary tap water. Concrete specimens of C30 target strength grade were prepared according to the Chinese standard JGJ55-2011, and the specific mix proportion of concrete is shown in Table 1. In the experiment, the slump of concrete was controlled in the range of 100 to 150 mm by adjusting the amount of water-reducing agent. According to the corresponding mix proportion of concrete, the slump of concrete mixture was 120 mm, which conformed to the Chinese standard JGJ55-2011.

### 2.2. Manufacture of Concrete Specimen

**Figure 1:**
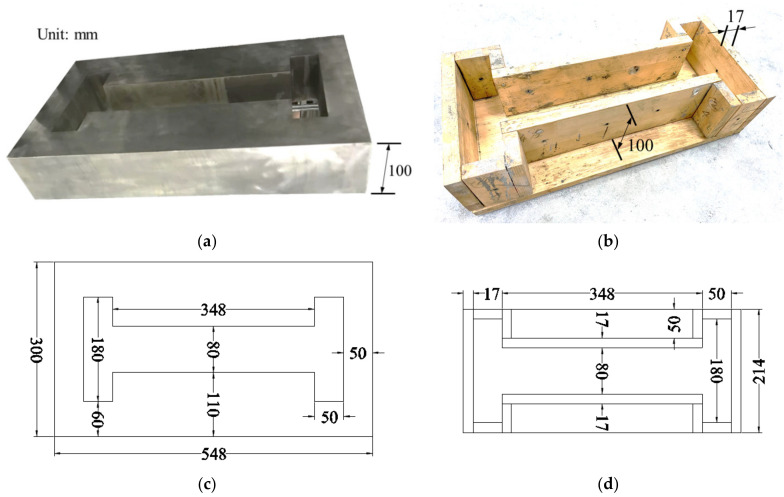
Restraint device, and non-restrained specimen mold. (**a**) Restrained device; (**b**) non-restrained specimen mold; (**c**) the specific dimensions of the top view of the restrained device; (**d**) the specific dimensions of the top view of the non-restrained specimen mold.

Invar, as a nickel–iron alloy, has a very low coefficient of thermal expansion and can maintain a fixed length in a wide temperature range. Taking Invar alloy as raw material, a relevant restraint device that meets the experimental conditions was designed and manufactured, as shown in Figure 1a, and the non-restrained specimen mold is shown in Figure 1b. The height of the concrete specimens was designed to be 100 mm, and the thickness of all wood panels was 17 mm. The specific dimensions of the top view of the restrained device and mold are shown in Figure 1c,d, respectively. The restrained and non-restrained specimens were poured according to the designed mix proportion.

### 2.3. Environmental Condition

The experimental environment was controlled by a high–low temperature alternating temperature humidity test chamber. The measured temperature range was −20 to 150 °C, and the relative humidity range was 30% to 98%.

**Figure 2:**
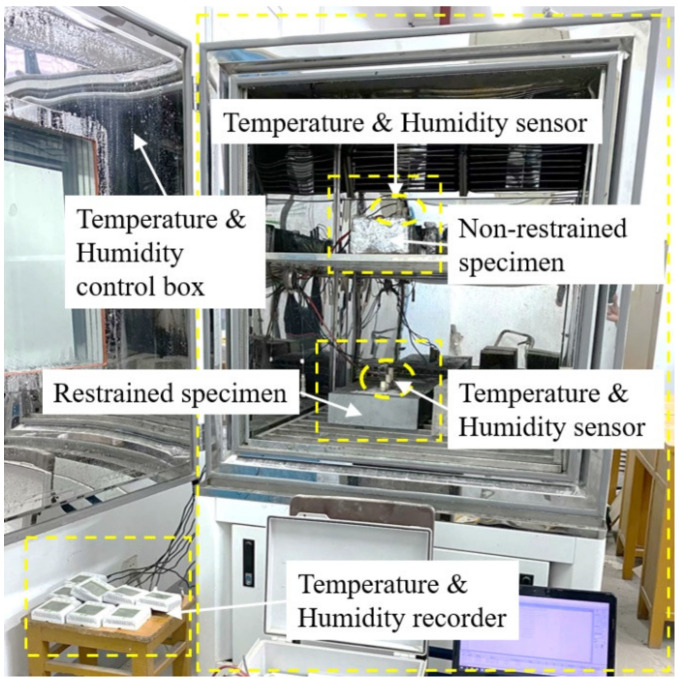
The overall effect of the experiment.

The digital signal of the transmitter was collected regularly by the inspection instrument and stored by the computer. The experimental environment temperature was set at 50 °C, the relative humidity was set at 30% and the loading time was 7 days. The experiment was carried out after the specimen was ready for pouring. The overall effect of the experiment is shown in Figure 2.

### 2.4. Experimental Process

In order to ensure the one-dimensional transmission of moisture along the height direction of the specimen, tin foil paper and oil paper were laid on the inner surface and bottom of the restraint device and the non-restrained specimen mold, and a layer of waterproof coating was brushed onto these. For the non-restrained specimen after mold removal and sealing with tin foil wrapping, all specimens were only left on the upper surface in contact with the air.

**Figure 3:**
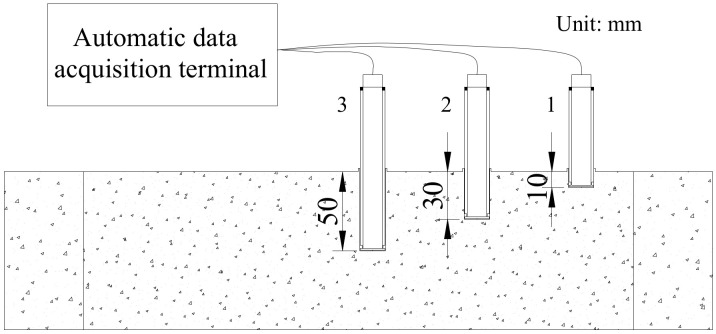
Sensor embedding mode.

The plastic tubes for placing the sensor with an outer diameter of 20 mm were prepared. Two segments of a 3 mm-wide unconnected annular belt along the outer ring line 2 mm away from the bottom of the tube were cut, and the bottom of the plastic tube was sealed with a plastic sheet. The stainless steel bars with a diameter of 18 mm were prepared and placed in the plastic pipe in advance, and the inner wall of the plastic pipe was kept in close contact with the steel bar. The length of the steel bar exceeded the upper end of the plastic pipe by at least 5 cm for easy removal. The purpose was to ensure that the cement slurry did not penetrate from the bottom hole during the vibration process. The length of the sensor probe was 3 cm, and two O-shaped rubber sealing rings were sleeved at the position of 3 cm. Then, the mixed concrete was poured into the mold and fully vibrated with a vibrating bar. Finally, the plastic tube with a steel bar was slowly inserted into the corresponding position of the concrete specimen. According to the above steps, three plastic pipes were inserted into each concrete specimen, and their depths in the concrete were 1 cm, 3 cm and 5 cm, as shown in Figure 3.

The steel bar was slowly pulled out after the pouring was completed for a period of time. The slurry remaining at the bottom of the plastic tube was sucked out with a sponge, and then the sensor was placed in the plastic tube, where the top of the sensor probe was as close as possible to the bottom of the plastic tube. As there was a gap between the outer wall of the sensor and the plastic tube, in order to ensure the accuracy of the collected humidity, a 2 mm-thick O-ring was placed 3 cm above the sensing part of the sensor so that the bottom of the plastic tube formed a sealed space of 3 cm in length. At the same time, the gap between the sensor and the PVC pipe was filled with polymer liquid sealant at the top of the plastic pipe to ensure that the sensor could accurately measure the temperature and humidity at the specified position. Then, the prepared concrete specimens were placed in the environmental test chamber, the environmental temperature was set at 50 °C and the relative humidity was set at 30%. The temperature and humidity were collected, and the data were recorded every 1 min.

### 2.5. Quantitative Characterization of Concrete Damage

**Figure 4:**
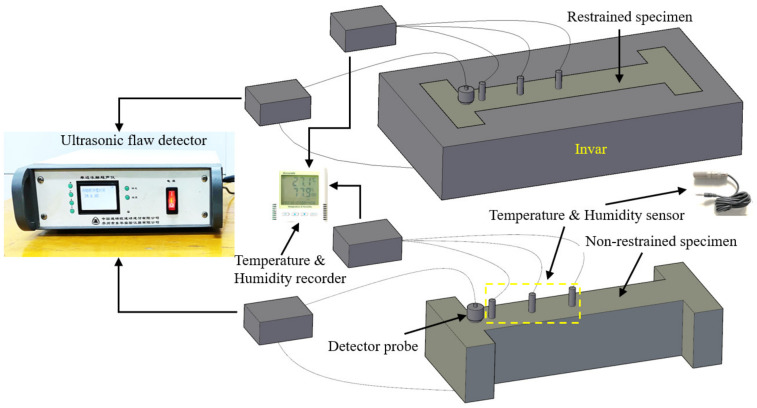
Quantitative characterization of damage of different concrete specimens.

The principle of ultrasonic flaw detection [35] was used to quantify the damage development of concrete specimens. The propagation time of the concrete specimens on the 0th, 1st, 3rd, 5th and 7th days of the loading age was measured by using a unilateral freeze–thaw ultrasonic flaw detector. The measurement position corresponds to the center of the diagonal at one end of the concrete specimen. Quantitative characterization of damage of different concrete specimens is shown in Figure 4. The measurement method was the opposite method, and the average value was taken and recorded three times each time. In order to minimize the acoustic energy loss, white Vaseline was used as a coupling agent. Based on the propagation time obtained by ultrasonic testing, the damage variable D was established. Therefore, the definition of D is given as

(1) $$D=1-{\left(\frac{t_{0}}{t_{i}}\right)}^{2}$$

where $t_{i}$ is the propagation time corresponding to the concrete specimen at time i, and $t_{0}$ is the propagation time corresponding to the concrete specimen at the initial time.

## 3. Results and Analysis

### 3.1. Changes in Concrete Temperature and Relative Humidity

**Figure 5:**
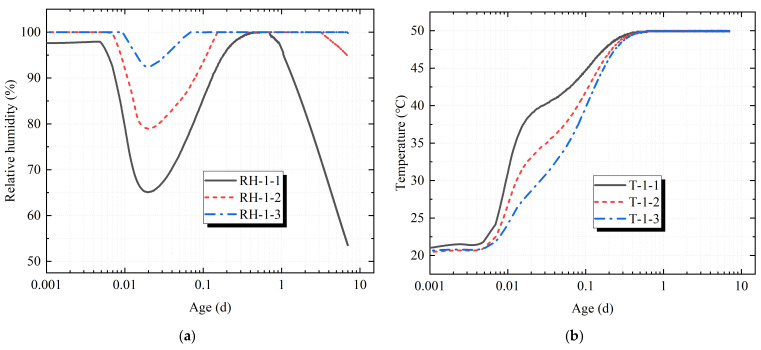
Duration curve of restrained specimen. (**a**) Duration curve of temperature; (**b**) duration curve of relative humidity.

**Figure 6:**
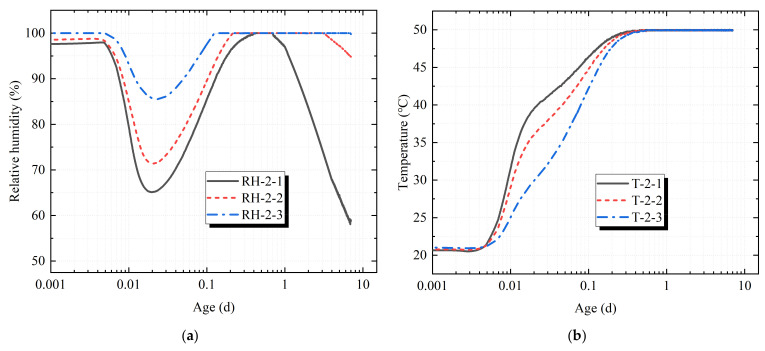
Duration curve of non-restrained specimen. (**a**) Duration curve of temperature; (**b**) duration curve of relative humidity.

In order to distinguish the temperature and humidity collected by the sensors, the sensor numbered 1 for each specimen was named as temperature sensor T-1-1 and humidity sensor RH-1-1 in this paper, and the sensors numbered 2 and 3 were also named in this way. It can be seen in Figure 5 and Figure 6 that the temperature set in the test chamber was 50 °C, and the concrete specimen placed in the test chamber just after pouring was affected by its own cement hydration heat release and higher ambient temperature. The concrete temperature response rate measured by the temperature sensors T-1-1, T-1-2, T-1-3, T-2-1, T-2-2 and T-2-3 was faster at the start time of the test chamber. The depth of T-1-1 and T-2-1 was the closest to the surface of the concrete, and the response rate was the fastest. Sensors T-1-2 and T-2-2 took second place, and sensors T-1-3 and T-2-3 were the slowest with the increase in the hole depth. The temperature measured by sensor T-1-3 fluctuated to a certain extent around the 0.6th day of the loading age, but the temperature eventually rose rapidly to 50 °C and then remained stable.

The relative humidity in the test chamber was set at 30%. The relative humidity measured by the sensor first decreased rapidly, then increased rapidly, and then decreased slowly, with large fluctuations. The main reason was that at the start-up time of the machine, the humidity in the hole was first measured by the sensor. Under the action of high temperature, the wet air evaporated and dissipated, and the relative humidity measured by the sensor decreased rapidly. On the other hand, as the initial stage of concrete pores contained more free water, they were in a state of humidity saturation. Under the action of high temperature, the free water inside the concrete began to evaporate, and the water vapor continued to attach to the sensor, resulting in the relative humidity measured by the sensor remaining at nearly 100% for a period of time. From then, under the continuous action of the high-temperature and low-humidity environment, the relative humidity measured by the sensor gradually decreased. The main reason is that the concrete cement itself had a certain degree of hydration and formed a certain amount of particles. When the free water was gradually isolated by the hydrated particles, and its continuity was destroyed, the vapor pressure in the pore began to be lower than the saturated vapor pressure. While the free water in the pore of the concrete decreased, the capillary water and gel water in the concrete began to evaporate continuously, resulting in the relative humidity measured by the sensor decreasing gradually.

As sensors RH-1-1 and RH-2-1 were the closest to the upper surface of the concrete, the relative humidity measured by sensors RH-1-1 and RH-2-1 began to decrease on the 0.65th day and the 0.7th day of the loading age, respectively, and the relative humidity decreased the fastest. Sensors RH-1-2 and RH-2-2 took second place, and sensors RH-1-3 and RH-2-3 were the slowest with the increase in the hole depth. The relative humidity measured by sensors RH-1-1 and RH-2-1 decreased to 53.5% and 58.8%, respectively, on the 7th day of the loading age, and the relative humidity difference between them was 5.3%. It was speculated that the microcracks generated by the restrained concrete specimen caused by the restraint effect accelerated the water loss in the concrete. The relative humidity measured by sensors RH-1-2 and RH-2-2 began to decrease on the 3.10th day and on the 3.25th day of the loading age, respectively. On the 7th day of the loading age, the relative humidity measured by sensors RH-1-2 and RH-2-2 decreased to 94.8% and 95.1%, respectively. For sensors RH-1-3 and RH-2-3, the relative humidity measured on the 7th day of the loading age was still close to 100%. It can be seen that the change in the relative humidity in the concrete was an extremely slow process, and the response rate of the humidity in the concrete was much slower than that of the temperature.

### 3.2. Change in Concrete Damage

The measured data of the propagation time of the restrained concrete specimen (specimen 1) and non-restrained concrete specimen (specimen 2) on the 0th, 1st, 3rd, 5th and 7th days of the loading age were sorted. The damage variable D value of each specimen was calculated by Equation (1), as shown in Table 2.

**Figure 7:**
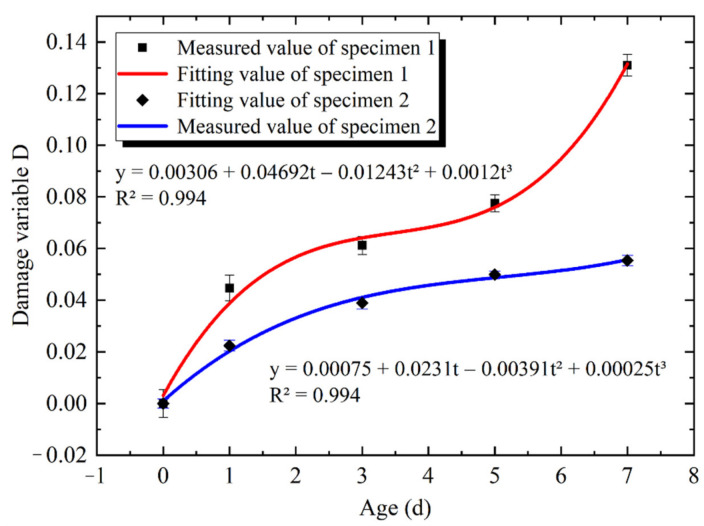
Comparative relationship between damage variable D and loading age t (d).

The relationship between the damage degree D and the loading age t (d) of specimens 1 and 2 was fitted by a cubic curve model [36]. The quantitative relationship between the damage degree D and the loading age t (d) was obtained, as shown in Equations (2) and (3). The comparison between the damage degree D and the loading age t (d) is shown in Figure 7. The details are as follows:

y = 0.00306 + 0.04692t − 0.01243t^2^ + 0.0012t^3^ (2)

y = 0.00075 + 0.0231t − 0.00391t^2^ + 0.00025t^3^ (3)

where t (d) is the loading age, and y is the damage variable D corresponding to the loading age t (d).

It can be seen in Figure 7 that the fitting value of the cubic curve model is in good agreement with the measured value. The damage degree D values of specimen 1 and specimen 2 increased with the increase in the loading age t (d), and the growth rate of the damage degree D value of specimen 1 was relatively fast. The difference in the D value between specimen 1 and specimen 2 was 0.02 on the 1st day of the loading age, and 0.07 on the 7th day of the loading age. The difference almost increased with the increase in the loading age. It can be seen that under the condition of high temperature and low humidity, the restraint increased the damage of concrete specimens.

### 3.3. Microscopic Morphology Observation

**Figure 8:**
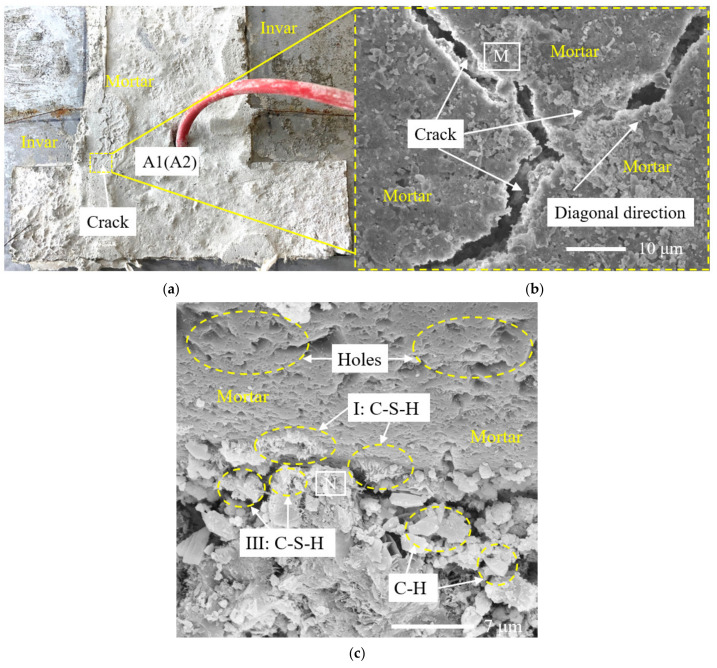
Microstructure and position of specimen 1. (**a**) Position of samples A1 and A2; (**b**) the microstructure of sample A1 and the position of region M; (**c**) the microstructure of sample A2 and the position of region N.

**Figure 9:**
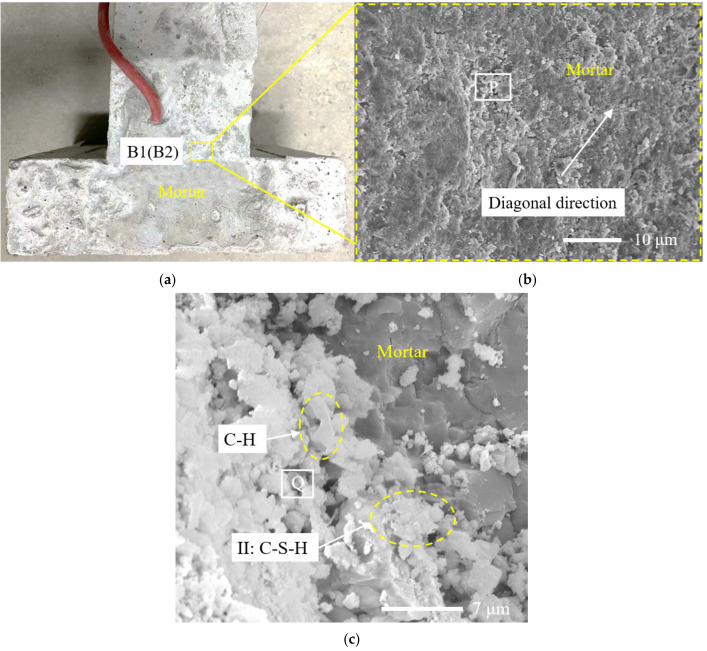
Microstructure and position of specimen 2. (**a**) Position of samples B1 and B2; (**b**) the microstructure of sample B1 and the position of region P; (**c**) the microstructure of sample B2 and the position of region Q.

It can be seen in Chapter 3.1 that when the ambient temperature was 50 °C and the relative humidity was 30%, the relative humidity measured by the sensors RH-1-3 and RH-2-3, whose position depth was 30 mm from the upper surface, was close to 100% on the 7th day of the loading age. In order to make the results more obvious, samples A1 and A2 of specimen 1 (the position depth was 10 mm and 30 mm from the upper surface, respectively, and the specific position is shown in Figure 8a) were selected and observed by SEM microscopic morphology. Samples B1 and B2 of specimen 2 (the position depth was 10 mm and 30 mm from the upper surface, respectively, and the specific position is shown in Figure 9a) were also selected and observed by SEM microscopic morphology.

It can be seen in Figure 8a and Figure 9a that under the condition of high temperature and low humidity, both specimen 1 and specimen 2 show the phenomenon of surface whitening. For sample A1, as shown in Figure 8a, a small amount of microcracks appeared near the right angle of the Invar alloy restraint device on the 7th day of the loading age of high temperature and low humidity, as shown in Figure 8b. There was little difference between the morphology of other surface areas of specimen 1 and that of the concrete surface area of specimen 2. For sample B1, as shown in Figure 9b, the interface of the cement paste on the concrete surface was well bonded, and no microcracks were observed.

Diamond, S. divided C-S-H into four types: I, II, II and IV, according to the morphology [37]. It can be seen in Figure 9c that sample B2 was unevenly distributed, with a certain amount of hydration products, and there were lamellar CH crystals with good crystallization. A type II C-S-H gel was uniformly distributed in all of the hydration products, and clusters were distributed. There were gaps between each cluster. The sizes of various hydration products were small, and the overall structure was relatively dense.

It can be seen in Figure 8c that the microstructure of sample A2 and sample B2 was similar, but there were still some differences. After heating, the cement hydration products of sample A2 had fewer lamellar CH crystals, and the rod and needle were slightly coarser than those of sample B2, resulting in a flocculent type I C-S-H gel, and a large and irregular type III C-S-H gel. The gels were interspersed and unevenly distributed, and there were pits and loose pores on the surface of the structure. It can be seen that the distribution uniformity of the hydration products of sample A2 on the 7th day of the loading age under high temperature and low humidity was worse than that of sample B2.

The reason is that under the continuous action of environmental conditions with a temperature of 50 °C and relative humidity of 30%, the free water in the pores of the concrete decreased, while the capillary water and gel water in the concrete began to evaporate, the slurry continued to harden and shrink and the damage continued to increase. The microcracks formed by the interface between the aggregate and aggregate or between the aggregate and slurry in the concrete also increased, and for the restrained specimen, the restraint effect limited the drying shrinkage of the concrete, which would eventually lead to the aggravated generation and expansion of concrete microcracks perpendicular to the restrained direction, which also verifies the speculation in Section 3.1 and explains the cracking reason in Figure 8b. On the other hand, the loss of water led to the uneven hydration reaction of cementitious materials, and the hydration products were partially wrapped around the incomplete hydration part, resulting in a further incomplete hydration reaction, which would eventually lead to the hydration products of concrete in specimen 2 being lower than those in specimen 1.

It can be seen that under the environmental conditions with a temperature of 50 °C and relative humidity of 30%, the restraint effect was to intensify the formation of microcracks, affect the hydration of the cement at the micro level and, finally, increase the risk of concrete cracking perpendicular to the restrained direction at the macro level.

### 3.4. EDS Results and Analysis

**Figure 10:**
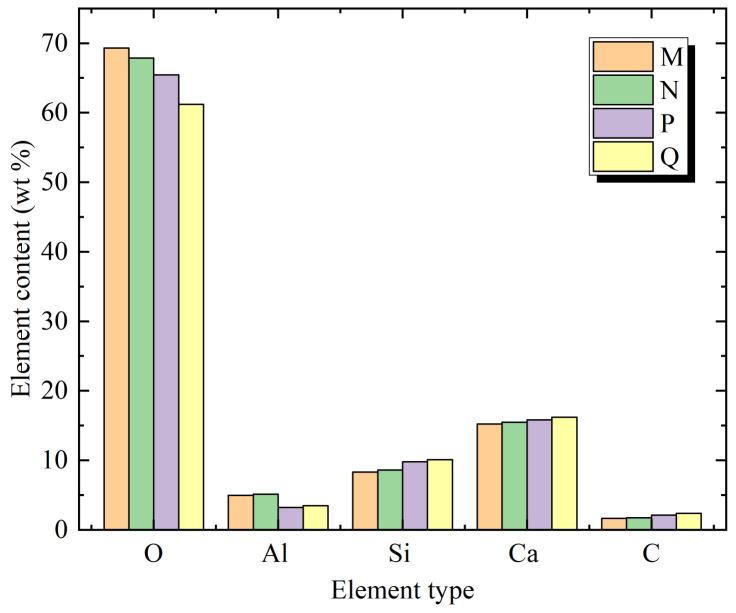
Comparison of EDS elements in different regions of concrete specimen samples.

Research has shown that Ca/Si could be used as the evaluation standard of the concrete hydration crystallization degree [38]. The Ca/Si of traditional Portland cement concrete was between 1.5 and 2.0, and most cases were close to 1.5. The comparison of EDS elements in different regions of concrete specimens is shown in Figure 10.

It can be seen in Figure 10 that for samples B1 and B2, the hydration products of regions P and Q were composed of O, Al, Ca, Si and C elements on the 7th day of the loading age. Except for the O element in region P being greater than that in region Q, other elements in region P were less than those in region Q. It can be inferred that there were hydration products such as C-S-H, CH, SiO_2_, Al_2_O_3_ and CaCO_3_. The Ca/Si ratios of P and Q were 1.62 and 1.61, respectively, which were slightly larger than 1.5. The crystallization degree was high, and the structural density was good. The Ca/Si ratio of region Q was 1.61, which was slightly smaller than that of region P, which was 1.62, indicating that the crystallization degree of region Q was slightly higher than that of region P.

It can be seen in Figure 10 that for samples A1 and A2, the hydration products of regions M and N were also composed of O, Al, Ca, Si and C elements on the 7th day of the loading age. Except for the O element in region M being greater than that in region N, other elements in region M were less than those in region N. It can be inferred that there were also hydration products such as C-S-H, CH, SiO_2_, Al_2_O_3_ and CaCO_3_ which were not significantly different from samples B1 and B2. The Ca/Si ratio of region N was 1.80, which was slightly smaller than that of region M, which was 1.83, indicating that the crystallization degree of region N was slightly higher than that of region M.

For Ca/Si in different regions of different samples, the Ca/Si in region M of sample A1 was higher than that in region P of sample B1, and the Ca/Si in region N of sample A2 was higher than that in region Q of sample B2. This also shows that the overall crystallinity of samples A1 and A2 was lower than that of samples B1 and B2 to some extent, which is consistent with the analysis results in Chapter 3.3.

### 3.5. XRD Results and Analysis

XRD is mainly used to analyze the crystal changes in a paste before and after the concrete hydration reaction, in order to qualitatively analyze the effect of the concrete hydration reaction. In order to further verify the results of the previous chapters, the samples A1, A2, B1 and B2 were further analyzed by XRD.

**Figure 11:**
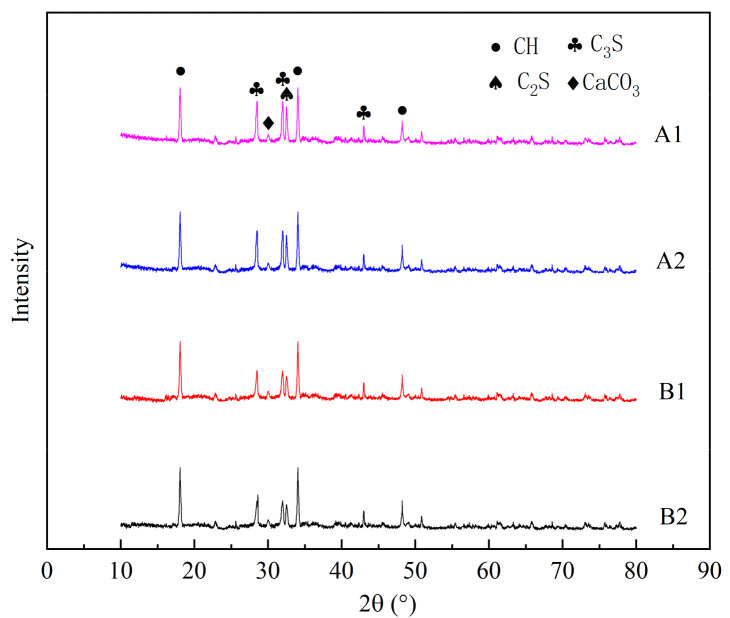
Comparison of XRD phases in concrete samples.

It can be seen in Figure 11 that the main phase compositions of hydration products of samples A1 and A2 were roughly the same as those of samples B1 and B2, which were mainly composed of hydration products CH and CaCO_3_. However, there were unhydrated cement clinker minerals C_3_S and C_2_S in samples A1, A2, B1 and B2. This shows that the main phase composition of hydration products of the concrete specimens was not changed by the restraint effect.

For samples A1, A2, B1 and B2, there were characteristic peaks of CH at 18.06θ, 34.06θ and 48.22θ, characteristic peaks of C_3_S at 28.5θ and 31.98θ, characteristic peaks of C_2_S at 32.5θ and characteristic peaks of CaCO_3_ at 30.02θ. Comparing the characteristic peaks of CH and CaCO_3_ of the four samples, the difference was relatively small, but the characteristic peaks of C_3_S and C_2_S of the A1 and A2 samples were higher than those of the B1 and B2 samples, respectively, and the difference was relatively obvious, indicating that the A1 and A2 samples contained more unhydrated cement clinker minerals, namely, C_3_S and C_2_S, which also shows that the hydration reaction effect of the A1 and A2 samples was not as good as that of the B1 and B2 samples, which is consistent with the results of the previous analysis.

It can be deduced that the formation of concrete hydration products in specimen 2 was a continuous and relatively stable process, and the formation process of concrete hydration products in specimen 1 was affected by the restraint effect.

## 4. Conclusions

In this paper, through the combination of macro experiments and micro-SEM, EDS and XRD detection methods, the following main conclusions were obtained for the early-age restrained concrete specimen under the environmental conditions of 50 °C and 30% relative humidity.

(a) The results show that the change in the relative humidity in the concrete was an extremely slow process, and the response rate of the humidity in the concrete was much slower than that of the temperature. The central temperature of the concrete specimen reached the maximum value of 50 °C around the 0.6th day of the loading age, and the central relative humidity of the concrete specimen was almost 100% on the 7th day of the loading age.
(b) The restraint increased the damage of the concrete specimens perpendicular to the restrained direction. When the central temperature of the specimen reached 50 °C, that is, at about the 0.6th day of the loading age, the difference in the damage degree between the restrained specimen and the non-restrained specimen began to become obvious, and the damage degree increased with the increase in the loading age. The relationship between the damage degree D and the loading age t (d) was fitted by a cubic curve model, and the quantitative relationship was obtained. The goodness of fit was 99.4%.
(c) The restraint intensified the generation and expansion of microcracks in concrete specimens and accelerated the loss of water in the concrete. On the 7th day of the loading age, the relative humidity decreased at the depths of 1 cm, 3 cm and 5 cm of the concrete specimens by 5.3%, 0.3% and 0.01%, respectively, under the restraint.
(d) The restraint effect manifested by intensifying the generation of microcracks and affecting the hydration of the cement at the micro level, resulting in a decrease in hydration products, a decrease in the crystallization degree and structural consistency density and, ultimately, an increase in the risk of concrete cracking at the macro level.
(e) The loading age from the 0.6th day to the 1st day was the period of a relatively large fluctuation in the concrete temperature and humidity, which might be the key period of concrete damage and microcrack aggravation. Therefore, it is necessary to perform a good job in moisture conservation measures of concrete surfaces at an early age.

## Figures and Tables

**Table 1:** Mix proportion of concrete.

Concrete Grade	w/c	Sand Content Rate	Water-Reducing Agent	Content/(kg·m^−3^)				
				Water	Cement	Sand	Aggregate I (5 to 10 mm)	Aggregate II (10 to 25 mm)
C30	0.47	0.419	2.1	175	370	780	324	756

**Table 2:** Damage variable D of concrete specimens.

The Loading Age (d)	0		1		3		5		7	
Concrete Specimen	1	2	1	2	1	2	1	2	1	2
Average transmission time (us)	35.4	35.6	34.6	35.2	34.3	34.9	34.0	34.7	33.0	34.6
Value of damage variable D	0	0	0.04	0.02	0.06	0.04	0.08	0.05	0.13	0.06

Note: According to Equation (1), the initial damage of each specimen was different, and the corresponding damage variables would be different for different specimens with the same propagation time.

## Data Availability

Data are contained within the article.
